# Small-molecule compound from AlphaScreen disrupts tau-glycan interface

**DOI:** 10.3389/fmolb.2022.1083225

**Published:** 2022-12-13

**Authors:** Shannon Faris, Weihua Jin, James Gibson, Anqesha Murray, Nathan Smith, Peng He, Fuming Zhang, Robert Linhardt, Chunyu Wang

**Affiliations:** ^1^ Department of Chemistry and Chemical Biology, Rensselaer Polytechnic Institute, Troy, NY, United States; ^2^ Center for Biotechnology and Interdisciplinary Studies, Rensselaer Polytechnic Institute, Troy, NY, United States; ^3^ Department of Biotechnology and Bioengineering, Zhejiang University of Technology, Hangzhou, China; ^4^ Department of Biological Sciences, Rensselaer Polytechnic Institute, Troy, NY, United States; ^5^ Department of Chemical and Biological Engineering, Rensselaer Polytechnic Institute, Troy, NY, United States

**Keywords:** drug discovery, tau, Alzheimer’s disease, AlphaScreen, heparan sulfate proteoglycan (HSPG), tauopathies, heparin

## Abstract

Tauopathies are neurodegenerative diseases characterized by intracellular abnormal tau deposits in the brain. Tau aggregates can propagate from one neuron to another in a prion-like manner, mediated by the interaction between tau and cell surface heparan sulfate proteoglycans. We developed an AlphaScreen assay, with His-tagged tau and biotinylated heparin, to represent the tau-HS interface to target the tau-glycan interface. Using our AlphaScreen assay, with a Z-factor of 0.65, we screened ∼300 compounds and discovered a small-molecule compound (herein referred to as A9), which can disrupt the tau-heparin interaction with micromolar efficacy. A9 also effectively inhibited heparin-induced tau aggregation in Thioflavin T fluorescence assays and attenuated tau internalization by H4 neuroglioma cells. These results strongly suggest that A9 can disrupt the tau-glycan interface in both *in vitro* molecular and cellular environments. We further determined that A9 interacts with heparin rather than tau and does so with micromolar binding affinity as shown by nuclear magnetic resonance and surface plasmon resonance experiments. A9 binds to heparin in a manner that blocks the sites where tau binds to heparin on the cell surface. These results demonstrate our AlphaScreen method as an effective method for targeting the tau-glycan interface in drug discovery and A9 as a promising lead compound for tauopathies, including Alzheimer’s disease.

## 1 Introduction

Tauopathies, including Alzheimer’s disease and frontotemporal dementia, are progressive neurodegenerative diseases characterized by the intracellular deposition of misfolded and aggregated microtubule-associated protein tau fibrils in the brain (Goedert, 2004). Under normal physiological conditions, intrinsically disordered tau binds to microtubules and promotes their stability (Goedert, 2004; Wang and Liu, 2008; Baker et al., 2021). Under pathological conditions, tau aggregates and forms neurofibrillary tangles (NFTs) (Dominguez-Meijide et al., 2020). Misfolded tau can propagate and spread tau pathology in a prion-like manner throughout the brain (Goedert et al., 2017; Mudher et al., 2017). In the prion model, tau aggregates are released from a donor cell into the extracellular space, which then bind to the cell surface and are endocytosed into a recipient cell. In the recipient cell, the internalized tau acts as a template to promote the misfolding of endogenous tau, seeding more NFTs (Stopschinski and Diamond, 2017). Although the exact mechanism of tau prion-like spread is still not fully understood, it is well established that tau recognition using heparan sulfate (HS) chains on heparan sulfate proteoglycans (HSPGs) on the cell surface is required for tau internalization (Holmes et al., 2013). Thus, HS and tau interaction is a crucial step in the prion-like spread of tau pathology and can be targeted for novel therapeutics for tauopathies like Alzheimer’s disease.

HSPGs are composed of HS, a linear glycosaminoglycan (GAG) chain, covalently linked to a core protein. Found virtually on all cell surfaces, HSPGs can bind to numerous proteins through HS (Ori et al., 2008). HS-protein binding is driven by electrostatic interactions, between the negative charges of the sulfate groups in HS, and the positively charged amino acids in the protein (Xu and Esko, 2014). A polydisperse structural analog of HS that has been frequently used as an HS mimetic due to its widespread availability is heparin (Mohamed and Coombe, 2017; Alavi Naini and Soussi-Yanicostas, 2018). Heparin differs from HS in that it has a higher sulfation level and a higher content of iduronic acid (Mohamed and Coombe, 2017).

Using heparin and tau to represent the tau-HS interface, we developed an AlphaScreen assay to screen for small-molecule inhibitors. AlphaScreen (Amplified Luminescent Proximity Homogenous Assay Screen) is a proximity-based bead assay used to identify small-molecule compounds that can disrupt macromolecular interactions (Yasgar et al., 2016). A pair of donor and acceptor beads are attached to molecules of interest through affinity tags. In our assay, His-tagged tau is captured by NTA-donor beads while biotinylated heparin is captured by streptavidin beads. When the beads are brought in proximity to one another through their native biological interaction, the excitation of the donor bead at 680 nm results in a singlet oxygen-mediated energy transfer to the acceptor bead and emission at 620 nm. When the interaction is blocked by an inhibitor, the fluorescence emission will be significantly reduced (Yasgar et al., 2016). This approach has been applied to target crucial interactions in a variety of diseases, such as Spike/ACE2 interaction in SARS-CoV-2 (Hanson et al., 2020) and RNA-protein interaction (Baker et al., 2021) in tauopathies.

In this study, we applied the AlphaScreen method to screen compounds that disrupt the tau-heparin interaction. We discovered a micromolar inhibitor of the tau-heparin interface termed A9. Surface plasmon resonance (SPR) and nuclear magnetic resonance (NMR) studies determined that A9’s aromatic regions bind to heparin with micromolar affinity. In addition, A9 inhibits heparin-induced tau aggregation and cellular uptake of tau with ∼10–20 μM efficacy. Thus, A9 is a promising lead compound for targeting the tau-glycan interface in the prion-like spread of tau pathology.

## 2 Materials and methods

The diverse small-molecule compounds used in the screening were provided by the National Cancer Institute (NCI)/National Institutes of Health (NIH) Developmental Therapeutics Program (DTP). Within the DTP’s Drug Synthesis and Chemistry Branch, the Diversity Set VI was selected based on their compound benchmarks. The compounds were supplied as 10 mM DMSO stocks in 96-well plates stored at -20°C and checked for purity *via* LC/MS spectroscopy.

Recombinant full-length tau (2N4R, aa 1–441) with and without an N-terminal 6xHis tag was expressed and purified as previously described (Despres et al., 2019). Briefly, full-length tau was expressed in *E. coli* strain BL21-DE3 cells and induced by 0.5 mM isopropyl β-d-1-thiogalactopyranoside (VWR, Radnor, PA). Cells were harvested and stored in -20°C for short-term or -80°C for long-term storage until purification. The cell pellets were resuspended in 50 mM Tris, 200 mM NaCl, pH 7.5, supplemented with 1.5X EDTA-free protease inhibitor tablets (Thermo Fisher, Waltham, MA) and 0.1 mM PMSF, then lysed by three passes through a microfluidizer at 80 psi. Cell debris was pelleted by centrifugation, and the supernatant was boiled in a water bath, chilled on ice for 5 min, and centrifuged. The supernatant was applied to a 5 mL HisTrap FF column (Cytiva, Marlborough, MA) and eluted using an imidazole gradient with final imidazole concentration at 350 mM. Fractions containing tau were pooled and dialyzed in 50 mM Na_2_HPO_4_, 1 mM EDTA, pH 6.5 and applied to a 5 mL SP FF column. Tau was eluted using NaCl gradient with final concentration of 1 mM. Fractions containing tau were pooled and concentrated, flash-frozen, and stored at -80°C.

Porcine intestinal heparin sodium USP lyophilized powder with an average molecular weight of ∼15 kDa, a polydispersity of 1.4 and an average sulfation degree of ∼3 sulfation per disaccharide unit purchased from Celsus Laboratories (Cincinnati, OH) was used in this study.

### 2.1 AlphaScreen assay

AlphaScreen assays were performed using OptiPlate-384 (PerkinElmer, Waltham, MA) flat white bottom plates. Reactants were diluted in a 1x AlphaLISA HiBlock Buffer (PerkinElmer) master mix (25 mM HEPES, 0.1% casein, 1 mg/mL Dextran-500, 0.5% Triton X-100, 0.5% Blocking reagent, 0.5% BSA and 0.05% Proclin-300, pH 7.4). AlphaLISA Streptavidin Acceptor beads, biotinylated heparin, and 6xHis tagged tau (His-tau) were added to the reaction master mix, resulting in final assay concentrations of 5 μg/mL, 0.1 μM, and 0.1 μM respectively. Addition of the AlphaScreen Nickel Chelate Donor beads was carried out in a dark room to minimize photobleaching due to photosensitivity of the beads, for a final assay concentration of 5 μg/mL. In each microplate well, assays were assembled by combining a fixed volume of A9 at varying concentrations with the master mix. The plate was sealed, shaken gently to mix the components thoroughly, and incubated for 1 h at room temperature in the dark. Laser excitations were carried out on a Tecan infinite M1000 Pro microplate reader equipped with AlphaScreen Assay software at 680 nm for 100 ms and with emissions recorded at 620 nm. Each AlphaScreen Assay sample was run in triplicate, including two controls, the compound solvent 1% DMSO as a negative (no inhibition) control and 10 μM heparin as a positive (inhibition) control. The data was fitted using log (A9) vs. response (as a three-way parameter) model on GraphPad v. 9.4.1 to obtain the IC_50_ with the equation Y=Bottom+Top−Bottom/1+10^X−LogIC50 . The Z-factor is defined as the ratio of the separation band to the signal dynamic range of the assay: $Z=1-\frac{\left(3\sigma_{s}+3\sigma_{c}\right)}{\left|\mu_{s}-\mu_{c}\right|}$ where σ_s_ and σ_c_ is the standard deviation of the samples (heparin) and controls (DMSO), respectively, and µ_s_ and µ_c_ denotes the mean of the samples and controls, respectively.

### 2.2 Surface plasmon resonance

*Preparation of heparin biochip.* Biotinylation of heparin was prepared similarly to our previous protocol (Kim et al., 2018). Briefly, heparin (2 mg) and amine–PEG3–Biotin (2 mg, Pierce) were dissolved in 200 μL of H_2_O and mixed with 10 mg of NaCNBH_3_. The reaction mixture was heated at 70°C for 24 h. This was followed by a further 10 mg addition of NaCNBH_3_, and the reaction was carried for another 24 h. After cooling to room temperature, the mixture was desalted with a spin column (3000 MWCO, Millapore Sigma, Burlington, MA). Biotinylated heparin was freeze-dried for chip preparation.

*Immobilization of biotinylated heparin onto SA chip.* The biotinylated heparin was immobilized on a streptavidin (SA) biochip (Cytiva, Uppsala, Sweden) based on the manufacturer’s protocol. In brief, 20 μL of solution of the heparin–biotin conjugate (0.1 mg/mL) resuspended in HBS-EP+ running buffer (0.01 M HEPES, 0.15 M NaCl, 3 mM EDTA, and 0.005% surfactant P20, pH 7.4) was injected over flow cells (FC) two, three and four of the SA chip at a flow rate of 10 μL/min. The successful immobilization of heparin was confirmed by the observation of an ∼150-resonance unit (RU) increase in the sensor chip. The control flow cell (FC1) was prepared by a 1 min injection with saturated biotin. The successful generation of the heparin biochip was confirmed by running full-length tau over the chip and comparing the binding kinetics to results from our previously published works (Zhao et al., 2020).

*Kinetic measurement of interaction between heparin and A9 using heparin biochip*. A9 was diluted in HBS-EP+ buffer (with 1% DMSO). Different concentrations of A9 (3.13, 6.25, 12.5, 25, 50, and 100 μM) were injected at a flow rate of 30 μL/min for 3 min on a Biacore T200 (Cytiva, Uppsala, Sweden). At the end of the sample injection, the same buffer was passed over the sensor surface to facilitate dissociation. After a 3 min dissociation time, the sensor surface was regenerated by injecting 30 μL of 2 M NaCl to obtain a regenerated surface. The response was monitored as a function of time (sensorgram) at 25°C. The sensorgram was fitted with a steady state affinity model (Biacore T200 Evaluation Software v. 3.0) (Cytiva, Uppsala, Sweden).

### 2.3 Nuclear magnetic resonance

Nuclear magnetic resonance (NMR) spectra of heparin and A9 were acquired at 20°C on a 600.13 MHz NMR spectrometer (Bruker, Billerica, MA) equipped with a cryogenic probe. Assignment of A9 was conducted through several ^1^H (1D), ^1^H-^13^C-heteronuclear single quantum coherence (HSQC), ^1^H-^1^H heteronuclear multiple bond correlation (HMBC), nuclear Overhauser effect spectroscopy (NOESY), and ^1^H-^1^H correlated spectroscopy (COSY) 2D experiments. Assignment of A9 was completed in 100% DMSO. In the titration experiments of A9 and heparin, a final solvent percentage of either 10/90% or 1/99% DMSO-d6/D_2_O was used. Assignment of A9 was performed in 100% DMSO while heparin titration into A9 was performed at 1% DMSO, due to the low solubility of heparin in DMSO. We determined that the percentage of DMSO influenced the chemical shifts of A9 in the ^1^H-^13^C HSQCs and ^1^H spectra. Therefore, we performed a DMSO titration into A9 to track these chemical shift perturbations, provided in the supplemental (Supplementary Figure S7).

A series of ^1^H and ^1^H-^13^C HSQCs were performed on a 1 mM heparin sample by adding in increasing amounts of A9 to final molar ratios of 1:1, 1:5, 1:50, and 1:100. Heparin and A9 were dissolved in 10/90% DMSO/D_2_O for the 1:1 M ratio titration experiments, 10/90% DMSO-d6/D_2_O for the 1:5 M ratio titration experiments and 1/99% DMSO-d6/D_2_O for the 1:50 and 1:100 M ratio titration experiments. All NMR data was processed and analyzed using Topspin 4.1.1 and NMRFAM Sparky (Lee et al., 2015).

### 2.4 Thioflavin T fluorescence assays

The kinetics and inhibition of tau were determined using Thioflavin T (ThT) fluorescence assays, performed on a Tecan Infinite M1000 microplate reader. A master mixture consisting of 10 μM His-tagged tau protein, 2.5 mM DTT, 10 μM heparin, and 10 μM ThT was freshly made in 10 mM HEPES, 100 mM NaCl, pH 7.4. Serial dilutions of A9 were used to measure the dose-dependent aggregation inhibition of tau protein. Control experiments were set up in parallel consisting of blank buffer with ThT, master mixture without heparin (“uninhibited tau”), the master mixture with 100 μM Tweezer (CLR01, a known inhibitor of tau aggregation (Sinha et al., 2011)) (“Tau + Tweezer”), and master mixture with 1% dimethyl sulfoxide (DMSO). Samples were tested and analyzed in quadruplicate. Protein aggregation was induced at 37°C with orbital shaking at 250 rpm for up to 60 h at an interval of 20 min. The ThT fluorescence intensity was monitored by exciting the molecule at 435 nm and recording the emission at 480 nm. Final data analysis and extraction of kinetic parameters were performed in Igor Pro program (Wavemetrics, Lake Oswego, OR).

Data was normalized to a scale of 0–1 with a baseline at 0 and the maximum of the lowest A9 concentration (0.017 μM) at 1. All aggregation curves are fitted to a sigmoidal function $s=base+\frac{\max}{1+\exp\left(\frac{x_{0}-x}{rate}\right)}$ to extract the half time of aggregation (*xhalf*)*, base, max* and *rate* where the coefficient *base* sets the Y value at small X. The Y value at large X is base + max. X_0_ sets the X value at which Y is at (base + max/s) and *rate* sets the rise rate. Smaller *rate* causes a faster rise, specifically, the slope at x = *x*_0_ is *max*/4*rate.

### 2.5 Cell culture and tau uptake assay

*Tau Protein Sample Preparation.* Upon tau purification, endotoxin removal was carried out using High-Capacity Endotoxin Removal Resin per manufacturer protocol (Thermo Fisher, Waltham, MA). Full-length tau was labeled with the Alexa Fluor 647 succinimidyl ester dye (AF-647) following manufacturer protocol (Thermo Fisher) resulting in degree labeling of 1.4 following PBS dialysis to remove excess dye. Labeled protein was stored at -80°C until use.

*Cellular Uptake Assays*. Human H4 neuroglioma cells purchased from ATCC were cultured in DMEM media supplemented with 10% FBS, and 100 μg/mL penicillin/streptomycin. Experiments were conducted at approximately 0.24 × 10^6^ cells/well confluency in a 24-well plate. Cultures were maintained in a humidified atmosphere of 5% CO_2_ at 37°C. H4 neuroglioma cells that were nuclei stained by Hoechst 33258 were introduced to A9 at 50, 25, 12.65, and 6.25 μM (each concentration with three technical replicates) followed by constant concentration of 0.1 μM tau labeled with Alexa Fluor 647 (tau-AF647) and incubated for 30 min at 37°C. Following incubation, the cells were washed with PBS and prepped for live cell imaging in DMEM media (without phenol red) supplemented with 5% FBS. Hoechst 33258 nuclei staining imaged under the DAPI channel while tau AF-647 was imaged under the CY5 channel on the EVOS M5000 fluorescent microscope. Calculation of the Corrected Total Cell Fluorescence (CTCF) for the tau cellular uptake assay microscopy images was completed using ImageJ and the formula $CTCF=Integrated\;Density–\left(Area\;of\;selected\;cell\;XMean\;fluorescence\;of\;background\;readings\right)$ . CTCF results were processed on Graphpad Prism v. 9.4.1. And a one-way ANOVA (Dunnett’s multiple comparisons test) was calculated, ns—not significant, ** - *p* < 0.001, **** - *p* < 0.0001.

## 3 Results

### 3.1 AlphaScreen assay identifies novel Tau-HS inhibitor compound

**FIGURE 1:**
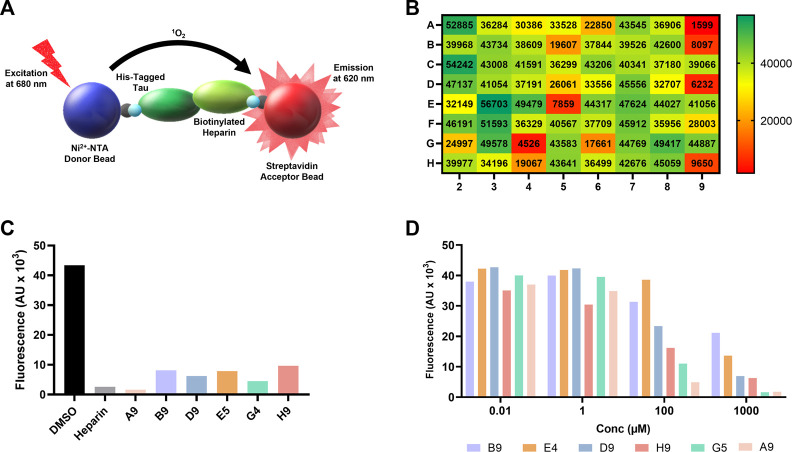
AlphaScreen of NCI compound library identifies several potential tau-glycan inhibitors**. (A)** Schematic of AlphaScreen assay demonstrating the interaction of the donor and acceptor beads by tau-heparin interaction, resulting in fluorescence emission. **(B)** Heatmap of NCI’s plate 4879 AlphaScreen fluorescence values colored from high fluorescence signal (i.e., no hit, dark green) to low fluorescence signal (i.e., positive hit, dark red). **(C)** Six lead compounds were identified, the fluorescent intensity of DMSO blank and heparin positive control compared to the six candidates. **(D)** AlphaScreen dose-dependent results of NCI’s plate 4879 top six hits compounds were diluted in series from 1,000 to 0.01 μM. Compound named corresponding to their respective position of the original NCI microplate.

FDA approved drug compounds that target the tau-heparin interface to slow the spread of tauopathies are lacking. We developed an AlphaScreen assay, in which we immobilized 6xHis-tagged full-length tau (His-tau) to nickel chelate (Ni^2+^-NTA) donor beads and biotinylated heparin to streptavidin acceptor beads, to address this issue (Figure 1A). For a high-throughput screening assay, the quality of the assay can be evaluated by the Z-factor (Zhang et al., 1999). If a screening assay has a Z-factor of 1, it is an ideal assay. If the value is found within 1 > Z ≥ 0.5, an assay is considered excellent (Zhang et al., 1999). The Z-factor for our assay was determined to be 0.65 (see Supplementary Table S1).

**FIGURE 2:**
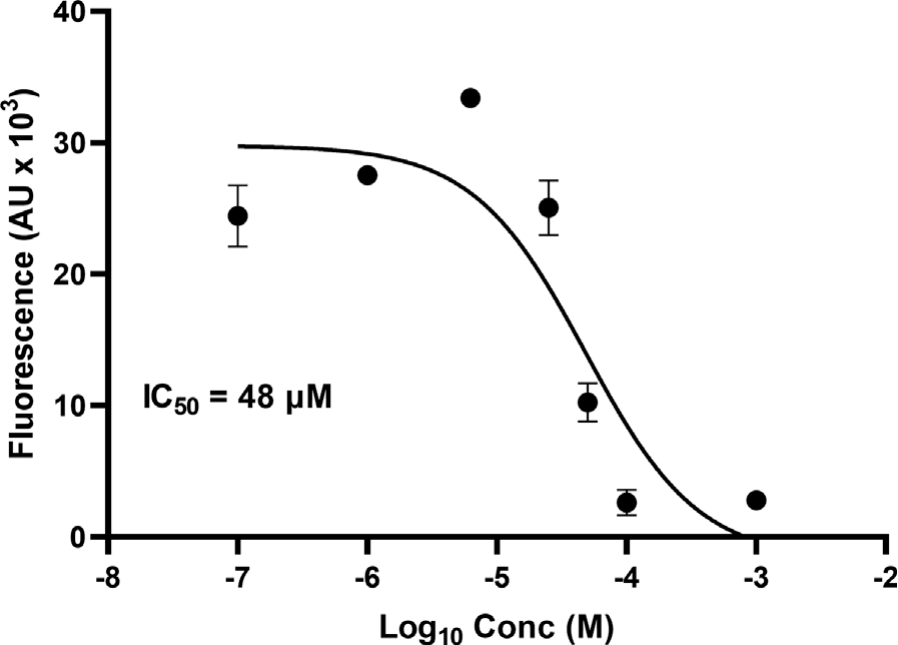
AlphaScreen IC_50_. IC_50_ of A9 determined to be 48 μM through dose-dependent AlphaScreen studies (in triplicate). *X*-axis is log [A9], and *Y*-axis is the fluorescence intensity (AU X 10^3^). IC_50_ derived from a three-way parameter model on GraphPad 9.4.1.

**FIGURE 3:**
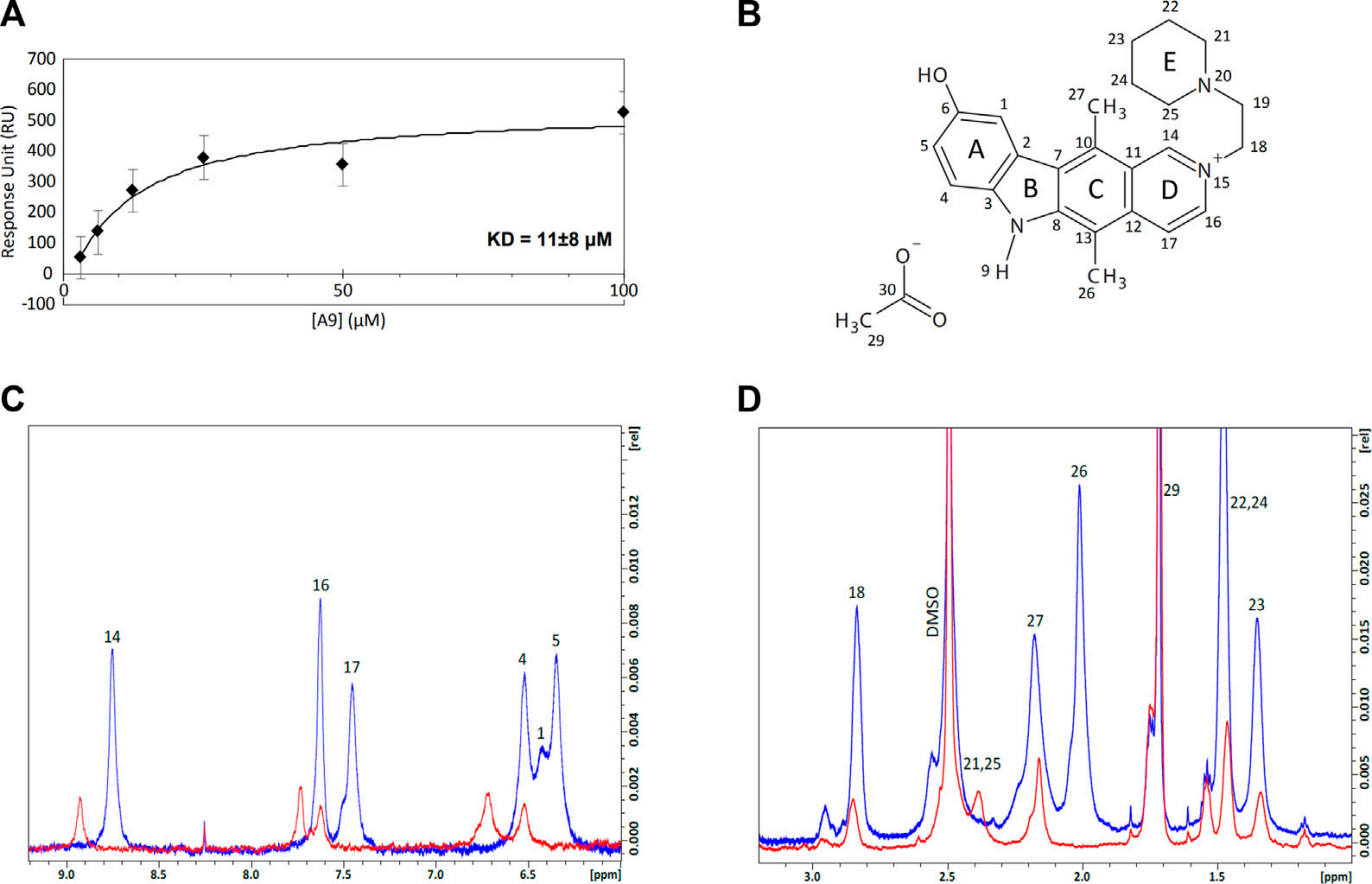
Equilibrium affinity and binding regions of A9 to heparin. **(A)** Equilibrium affinity of A9 binding to heparin determined by SPR to be 11 μM. A9 concentrations are 3.125, 6.25, 12.5, 25, 50, and 100 μM. The black curve represents the best fit of a steady state affinity model (Biacore T200 Evaluation Software v. 3.0). See Supplementary Figure S3 for all FCs. **(B)** Numbered structure of A9. Chemical names include: 9-hydroxy-2-(2-piperidinylethyl)ellipticinium acetate and 6H-Pyrido[4, 9-hydroxy-5,11-dimethyl-2-[2-(1-piperidinyl)ethyl]-, acetate. Molecular Formula: C24H28N3O.C2H3O2. Molecular Weight: 434.0 g/mol. PubChem SID: 573696. 92764817. **(C)** Overlay of the ^1^H spectra zoomed in to the aromatic region of A9 (6–9.2 ppm) by itself (blue) and in complex with heparin (red) at 100:1 M ratio. Complex formation shows loss of ^1^H signal and significant chemical shift perturbations (CSPs) from A9’s aromatic region in the presence of heparin. **(D)** Overlay of the ^1^H spectra zoomed in to the aliphatic region (1–3.2 ppm) of A9 by itself (blue) and in complex with heparin (red) at 100:1 M ratio. Complex formation shows loss of ^1^H signal from A9’s aliphatic region in the presence of heparin.

One microplate from the NCI diversity set (DTP library) was screened at a time. A total of ∼300 compounds were screened. All compounds were screened at 100 μM. Fluorescent signal results from each plate screened were visualized in a heat map to easily identify compounds with low fluorescent signal, which corresponds to inhibitors of the tau-heparin and potentially tau-HS interface (Figure 1B). Plate 4879 from NCI’s Diversity Set VI screening resulted in the identification of six lead compounds with the lowest AlphaScreen fluorescent signal (Figure 1B in red and Figure 1C). Structure of each of the six lead compounds are in Supplementary Figure S1. These compounds were probed further in a dose-response AlphaScreen (Figure 1D) which indicated that A9 is the best inhibitory compound among these six, with its structure shown in Figure 3B. We then proceeded to determine the IC_50_ of A9, by carrying out AlphaScreen over a wide range of concentrations. The IC_50_ of A9 was determined to be 48 μM (Figure 2).

### 3.2 A9 interacts with heparin at μM affinity

We employed Surface Plasmon Resonance (SPR) to define the kinetic parameters (k_on_, k_off_, K_D_) of the A9-heparin complex to determine the binding affinity of the A9-heparin interaction. A9 was diluted in HBS-EP+ buffer (with 1% DMSO) and injected in decreasing concentrations over a heparin immobilized sensor chip. The analyte A9 showed immediate saturation at 30 μL/min flow rate when interacting with the immobilized heparin, preventing the typical fitting of kinetic information to a 1:1 Langmuir model. Binding affinity was determined using a steady-state affinity equation whereby the resultant K_D_ of A9-heparin interaction was calculated to be 11 ± 8 μM (Figure 3A), which is within the same order of magnitude of the IC_50_ of 48 μM from AlphaScreen results. Due to the difficulty in obtaining a functional tau immobilized SPR chip, we used nuclear magnetic resonance (NMR) to probe the tau-A9 interface and found that A9 does not bind to tau (data not shown). Thus, A9 disrupts the tau-HS interaction by occupying the glycan instead of tau.

We titrated tau or heparin into A9 in NMR experiments to understand the basis of tau/glycan complex disruption by A9. First, A9 was assigned with ^1^H-^13^C heteronuclear single quantum coherence (HSQC), ^1^H-^1^H heteronuclear multiple bond correlation (HMBC), ^1^H-^1^H nuclear Overhauser effect spectroscopy (NOESY), and ^1^H-^1^H correlated spectroscopy (COSY) experiments (See Supplementary Material). ^1^H spectra were used to visualize the changes in the A9 signal upon titration of heparin (Figures 3C,D). At a 50:1 M ratio of A9 to heparin, no A9 peaks are visible in the ^1^H spectra, indicating that heparin had bound all the available A9 (data not shown). At a 100:1 M ratio of A9 to heparin, the overlay of the ^1^H spectra zoomed in to the aromatic region of A9 shows complex formation represented by the loss of ^1^H signal and significant chemical shift perturbations (CSPs) from A9’s aromatic region in the presence of heparin (Figure 3C). In contrast, overlay of the ^1^H spectra zoomed in to the aliphatic region of A9 shows complex formation represented by loss of ^1^H signal from A9’s aliphatic region in the presence of heparin, but not significant CSPs. This suggests that the major binding region of A9 to heparin is in the aromatic rather than the aliphatic region.

### 3.3 A9 inhibits heparin-induced tau aggregation

**FIGURE 4:**
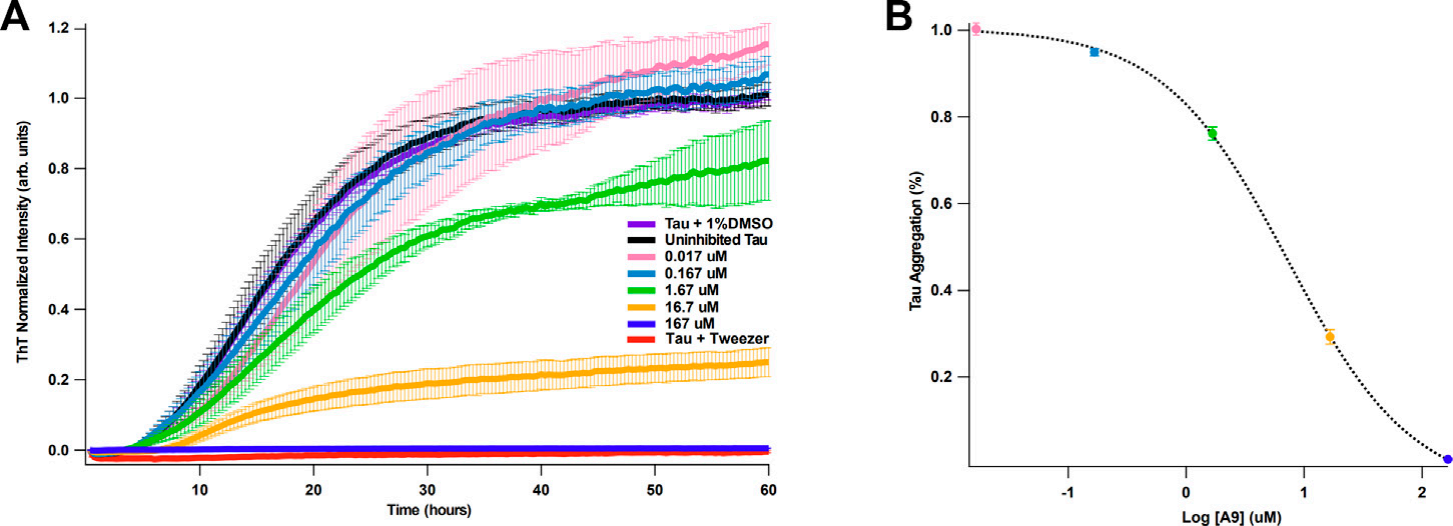
A9 inhibits heparin-induced tau aggregation with μM affinity. **(A)** Tau aggregation monitored by ThT fluorescence assay in the presence and absence of A9. The uninhibited tau aggregation control is shown in black, the inhibited tau aggregation control is shown in red, and the addition of 1% DMSO control is shown in purple. Inhibition of tau aggregation by varying concentrations of A9 are shown in pink, blue, green, orange, and navy (0.017, 0.167, 1.67, 16.7, and 167 μM, respectively). Tweezer was used a positive control for aggregation inhibition. Heparin at 10 μM was used across all samples to induce tau aggregation. **(B)** Plot of percentage tau aggregation inhibition as a function of A9 concentration (μM) in semi-log scale fitted to a sigmoidal model. The fitting was used to calculate the IC_50_ value of A9. Data is represented as mean ± standard deviation generated from three repeat measurements. The tau aggregation (%) data was normalized to the lowest concentration of A9 (0.017 μM).

It is well established that heparin can induce full-length tau aggregation (Goedert et al., 1996). Binding kinetics and affinity of tau and heparin were previously reported by our group: tau binds to heparin with a K_D_ of ∼20 nM (Zhao et al., 2020). We hypothesize that if A9 can interrupt tau-heparin interaction, A9 may reduce heparin-induced tau aggregation. Using a ThT fluorescence assay (Zhou et al., 2022), we demonstrate that A9 can inhibit heparin-induced tau aggregation in a dose-dependent manner (Figure 4A). A9 completely suppressed tau aggregation at 167 μM, the highest A9 concentration used (Figure 4A). As the A9 concentration decreased, the inhibition grew weaker, and tau aggregation became more prevalent. The IC_50_ of tau aggregation inhibition was determined to be 7.3 μM, which is in agreement with the K_D_ of 11 ± 8 μM from SPR for A9-heparin interaction (Figure 4B).

### 3.4 A9 inhibits tau internalization in H4 cells

**FIGURE 5:**
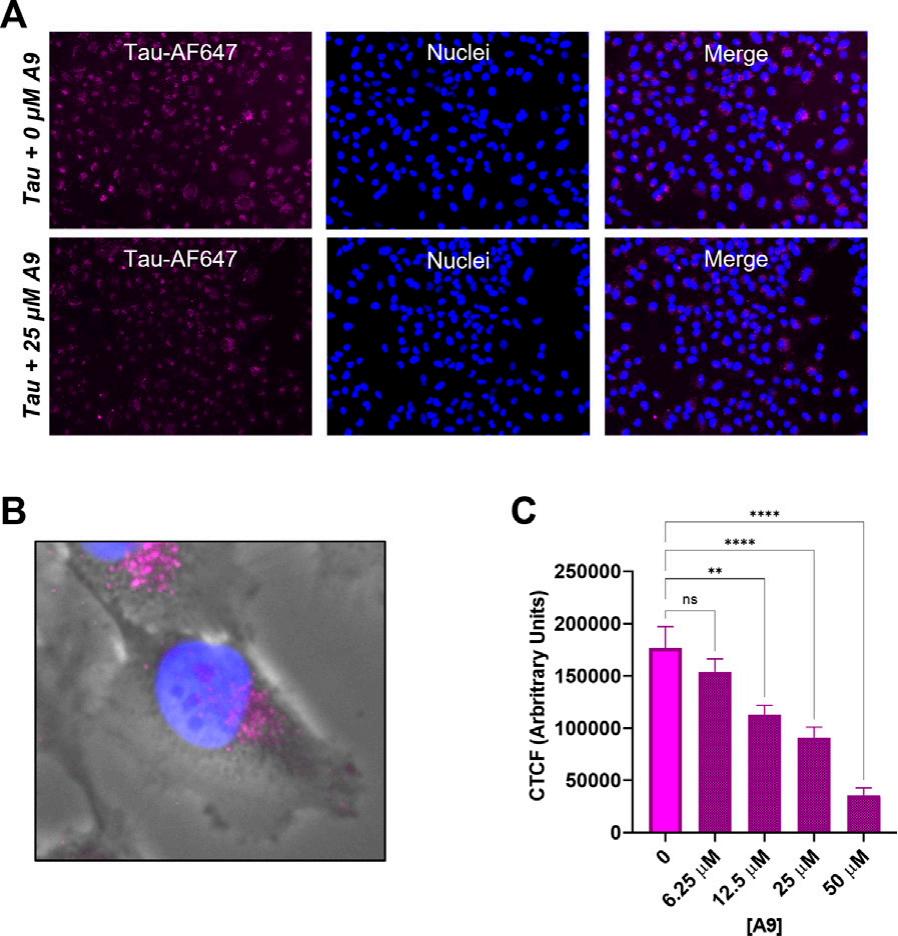
A9 reduces tau uptake in human H4 neuroglioma cells. **(A)** Microscopy images of tau uptake assay. H4 cells were treated with 0.1 μM tau-AF647 in the presence and absence of 25 μM A9. Tau-AF647 was imaged using the CY5 channel, nuclei were stained with Hoechst 33258 and imaged using the DAPI channel. **(B)** Magnification of a single cell showing tau uptake localized around the paranuclear region. **(C)** Quantification of 5A is reported as Corrected Total Cell Fluorescence (CTCF), which shows a dose-dependent inhibition of tau internalization by A9.

We next investigated whether A9 can inhibit the internalization of tau in a cellular environment. We carried out a tau cellular uptake assay using a human H4 neuroglioma cell line, which has been used for tau uptake assays (Rauch et al., 2020). HSPGs present on the cell surface of H4 neuroglioma cells are able to bind and internalize tau aggregates. The tau protein was labeled with Alexa-647 dye (tau-AF647) to track tau as it enters the cells. Tau-AF647 at 0.1 μM was used as a control to show uninhibited uptake of tau. H4 neuroglioma cells were first introduced to increasing concentrations of A9 followed by the addition of 0.1 μM tau-AF647. The cells were then incubated for 30 min at 37°C. At 25 μM A9, a clear inhibition of tau uptake was observed as compared to the tau control (Figure 5A).

A focused channel overlay image of a single cell reveals that tau is in endosomes around the paranuclear region, likely due to endocytosis (Figure 5B). Quantification of the corrected total tau fluorescence displayed a clear inhibition of tau internalization with increasing amounts of A9 present (Figure 5C). This result showed an approximate IC_50_ around the 25 μM concentration. The data strongly suggests that A9 inhibits tau uptake by blocking the interaction of tau with cell surface HPSGs.

## 4 Discussion

We report the identification of a small-molecule drug A9 (9-hydroxy-2-(2-piperidinylethyl)ellipticinium acetate) that is capable of inhibiting the tau-HS interface through a bead-based proximity HTS assay, AlphaScreen. From dose-dependent AlphaScreen experiments, A9 was determined to have an IC_50_ of ∼48 μM*.* Varying concentrations of A9 were flowed over a heparin sensor chip in SPR experiments to further understand A9 and its potential to disrupt the tau-HS interface. The equilibrium dissociation constant was determined to be (K_D_) to be 11 ± 8 μM.

We first performed various 1D and 2D NMR experiments to assign the molecule’s structure to investigate how A9 interacts with heparin at the molecular level. Post-assignment, we titrated A9 into heparin at 50:1 and 100:1 M ratios, the latter of which demonstrated an ∼50% peak intensity loss of A9 protons as well as significant chemical shift perturbations in the aromatic region. This strongly indicated that A9’s aromatic region binds to heparin rather than its aliphatic carbons, similar to the computational results of heparin and small molecules π-π stacking (Maszota-Zieleniak et al., 2021). Furthermore, the high ratio required to both visualize A9’s ^1^H signal indicate that the A9-heparin complex is not 1:1, and likely multiple A9 molecules bind to a single heparin chain. Presumably, this interaction occurs through electrostatic interactions, similar to how tau and heparin interacts. In the case of A9, the positively charged nitrogen could be interacting with the negatively charged sulfate groups on heparin. Additionally, hydrogen bonding can also be occurring in this interaction through the hydroxy group or the hydrogen on the nitrogen of A9. Although this is only a conjecture at this time, we have not yet investigated the specific binding interaction between A9 and heparin.

In a functional assay, it was shown that A9 can inhibit heparin-induced tau aggregation in a dose-dependent manner with an IC_50_ of 7.3 μM. In a cell uptake assay, utilizing H4 neuroglioma cells, with the introduction of A9 inhibited tau uptake in a dose-dependent manner. These data suggest that A9 can target multiple mechanisms of action (internalization, aggregation) dependent upon the tau-HS interaction.

The binding affinity of HS-tau interaction measured by SPR is ∼20 nM (Zhao et al., 2020), while K_D_ of heparin-A9 interaction is 11 μM measured here. To disrupt HS-tau interaction, higher concentrations of A9 will be needed compared with HS and tau. Accordingly, in our AlphaScreen assay, A9 is effective at disrupting tau-heparin interaction when present at ∼100-fold molar excess relative to the tau-heparin complex. Similarly, in our cellular uptake assay, the concentration of A9 used is much higher than the concentration of tau. These considerations highlight the need for additional screening efforts for a nM inhibitor.

In this study we used monomeric tau, instead of misfolded or aggregated tau, to represent the tau-HS interface in the prion-like spread of tau pathology. We justify the use of monomeric tau based on the fact that results obtained from monomeric tau are often applicable to tau aggregates. For example, the initial study demonstrating the importance of 6-O-sulfation in tau-HS interaction was carried out with monomeric tau, which have been later validated with tau aggregates (Zhao et al., 2017; Rauch et al., 2018; Stopschinski et al., 2018). In future studies, tau aggregates will be used in AlphaScreen and for the validation of hits.

Other work has been done in the field of tau-HS interface, such as a synthetic heparanoid that binds to tau and inhibits tau uptake and seeding in cells (Stopschinski et al., 2020). While their research focuses on heparinoids as drug candidates which binds to tau, our approach may yield compounds that interact with heparin. Here we present the characterization of a novel small molecule that binds to HS. Importantly, A9 serves as a strong chemical scaffold prime for optimization for HS binding. As an example, A9 could be optimized by changing the core pyrrolidine structure to a thiohydantoin. This change would mimic the core structure of a rhodamine-based compounds which is used in medicinal chemistry, specifically found to inhibit tau aggregation (Bulic et al., 2009). This opens a new avenue for therapeutic targets as there is a severe lack of effective treatments for tauopathies, including Alzheimer’s Disease.

## Data Availability

The original contributions presented in the study are included in the article/Supplementary Material, further inquiries can be directed to the corresponding author.
