# Supplementary material for: Small-molecule compound from AlphaScreen disrupts tau-glycan interface
Source: Front Mol Biosci. 2022 Dec 13;9:1083225. doi: 10.3389/fmolb.2022.1083225 (PMC9798536; doi:10.3389/fmolb.2022.1083225)
Supplement: Supplementary file 1 [file DataSheet1.docx]

Supplementary Material

## Supplementary Figures

| **Sample** | **Mean sample**  **(heparin, positive control for inhibition)** | **Mean control**  **(DMSO, negative control for inhibtion)** | **SD sample** | **SD control** | **Reported Z-factor** |
| --- | --- | --- | --- | --- | --- |
| **AlphaSignal** | **1461.5** | **43861** | **1089.5** | **3895** | **0.65** |

**Supplementary Table 1.** Reported value of standard deviation (SD) and means from AlphaScreen assay for the quantification of the Z-factor. $Z=1- \frac{\left( 3\sigma_{s}+3\sigma_{c} \right)}{\left| \mu_{s}-\mu_{c} \right|}$ here σ_s_ and σ_c_ is the standard deviation of the samples (25 μM heparin, positive control for inhibition) and controls (DMSO), respectively, and µ_s_ and µ_c_ denotes the mean of the samples and controls, respectively.

**
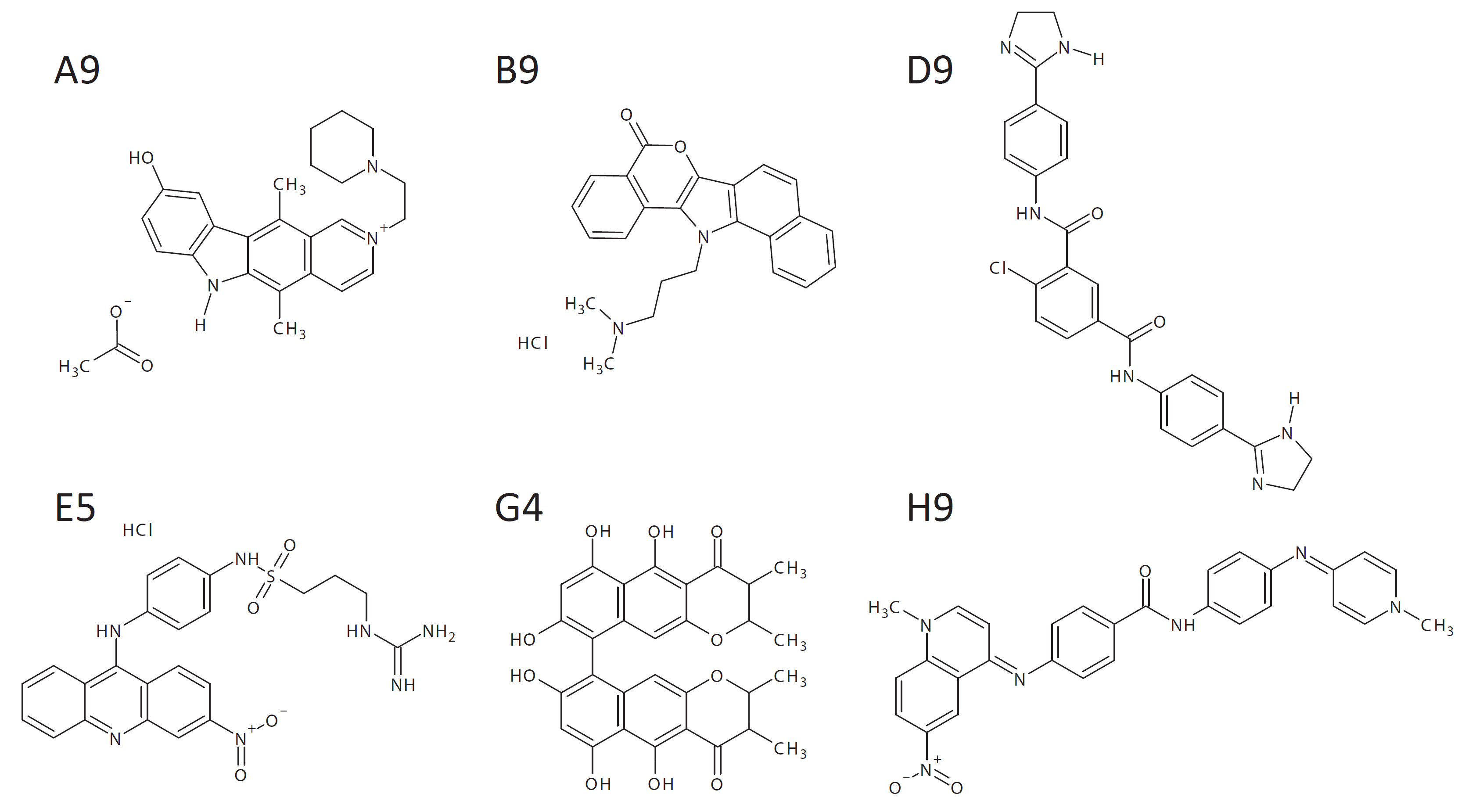
Supplementary Figure 1.** Chemical structures of the 6 lead compounds from the NCI diversity Set VI identified from the AlphaScreen assay. A9, B9, D9 *(top row from left to right)*, E5, G4, and H9 (*bottom row from left to right)*. Compounds each named corresponding to their respective positions of the original NCI microplate.


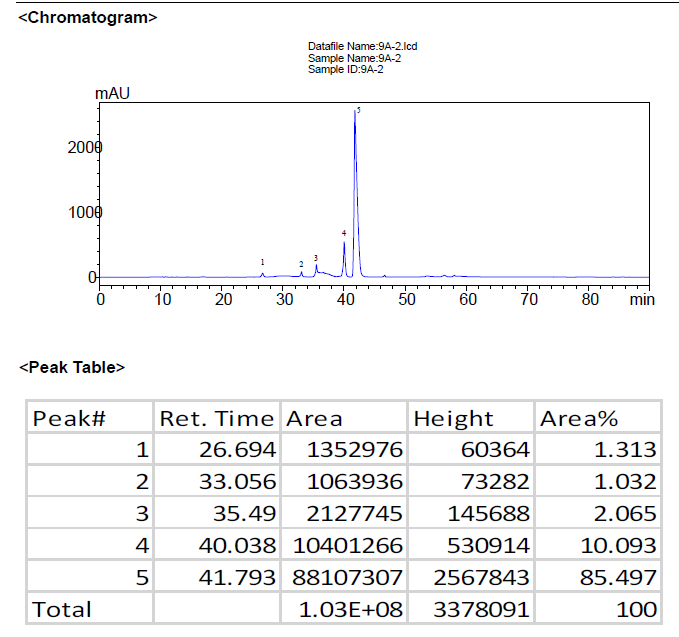
**Supplementary Figure 2.** HPLC results from purity check of A9.

| Peak # | Retention Time (min) | Area | Height | Area % |
| --- | --- | --- | --- | --- |
| 1 | 26.693 | 1352976 | 60364 | 1.313 |
| 2 | 33.056 | 1063936 | 73282 | 1.032 |
| 3 | 35.49 | 2127745 | 145688 | 2.065 |
| 4 | 40.038 | 10401266 | 530914 | 10.093 |
| 5 | 41.793 | 88107307 | 2567843 | 85.497 |
| Total |  | 1.03E+08 | 3378091 | 100 |

**Supplementary Table 2.** Peak table from HPLC chromatogram (Supp Fig 2).

**
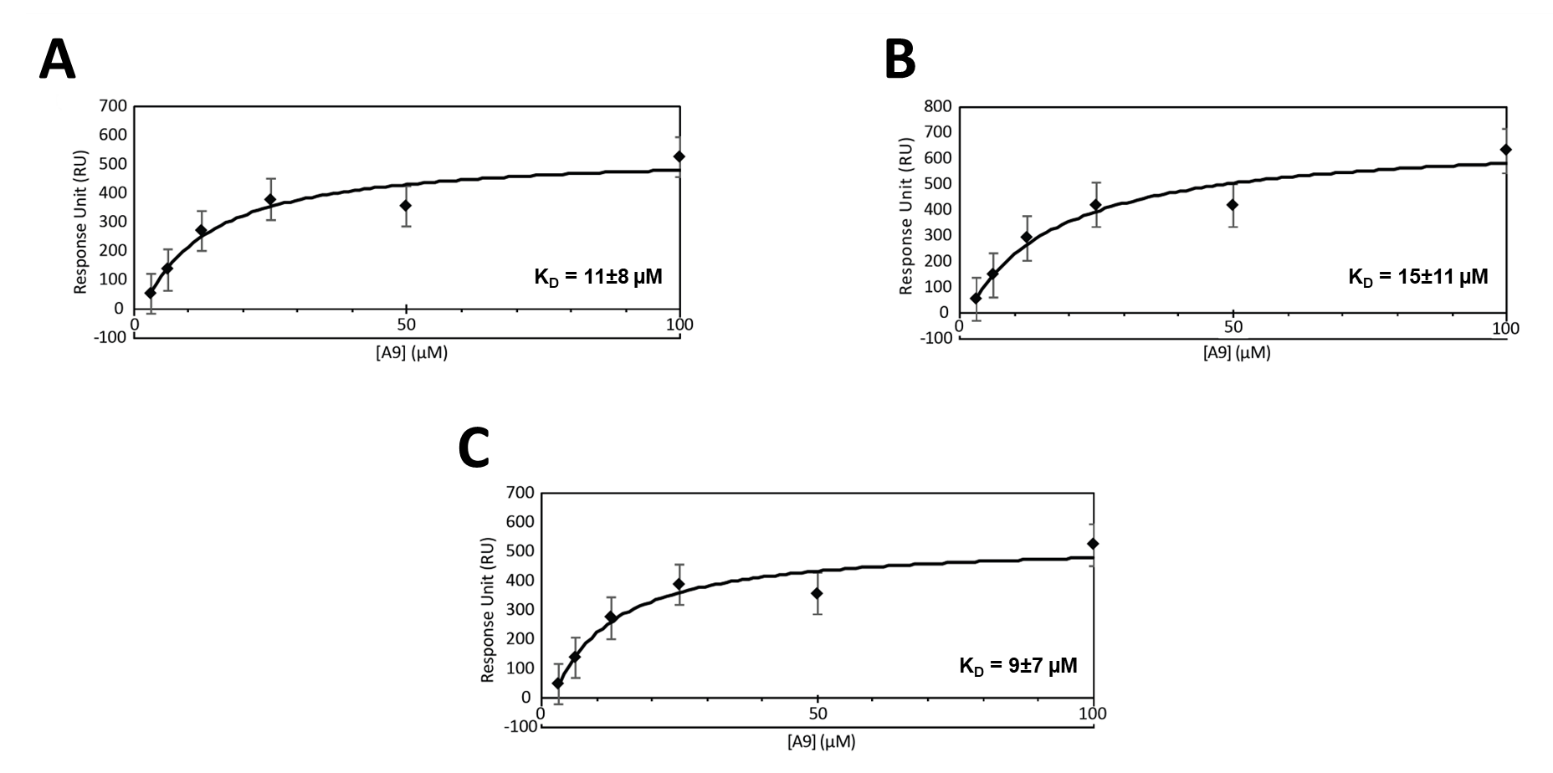
Supplementary Figure 3.** SPR binding isotherms of all reference subtracted FCs. (A) FC2-1. (B) FC3-1. (C) FC4-1. Note that FC2,3, and 4 have immobilized biotinylated heparin whereas FC1 has only biotin immobilized and serves as the reference lane to remove the influence of non-specific binding.


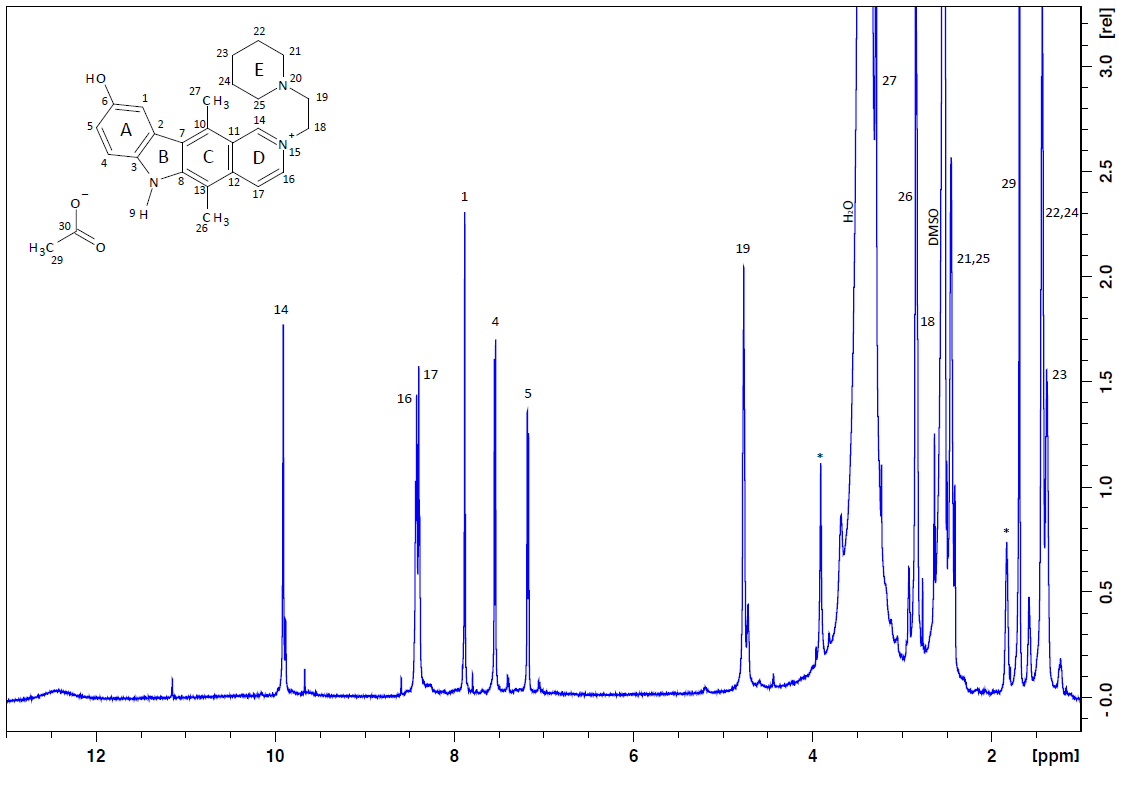
**Supplementary Figure 4.** ^1^H spectra of A9 in 100% DMSO with assignment. Asterisk (*) indicates impurity peak.


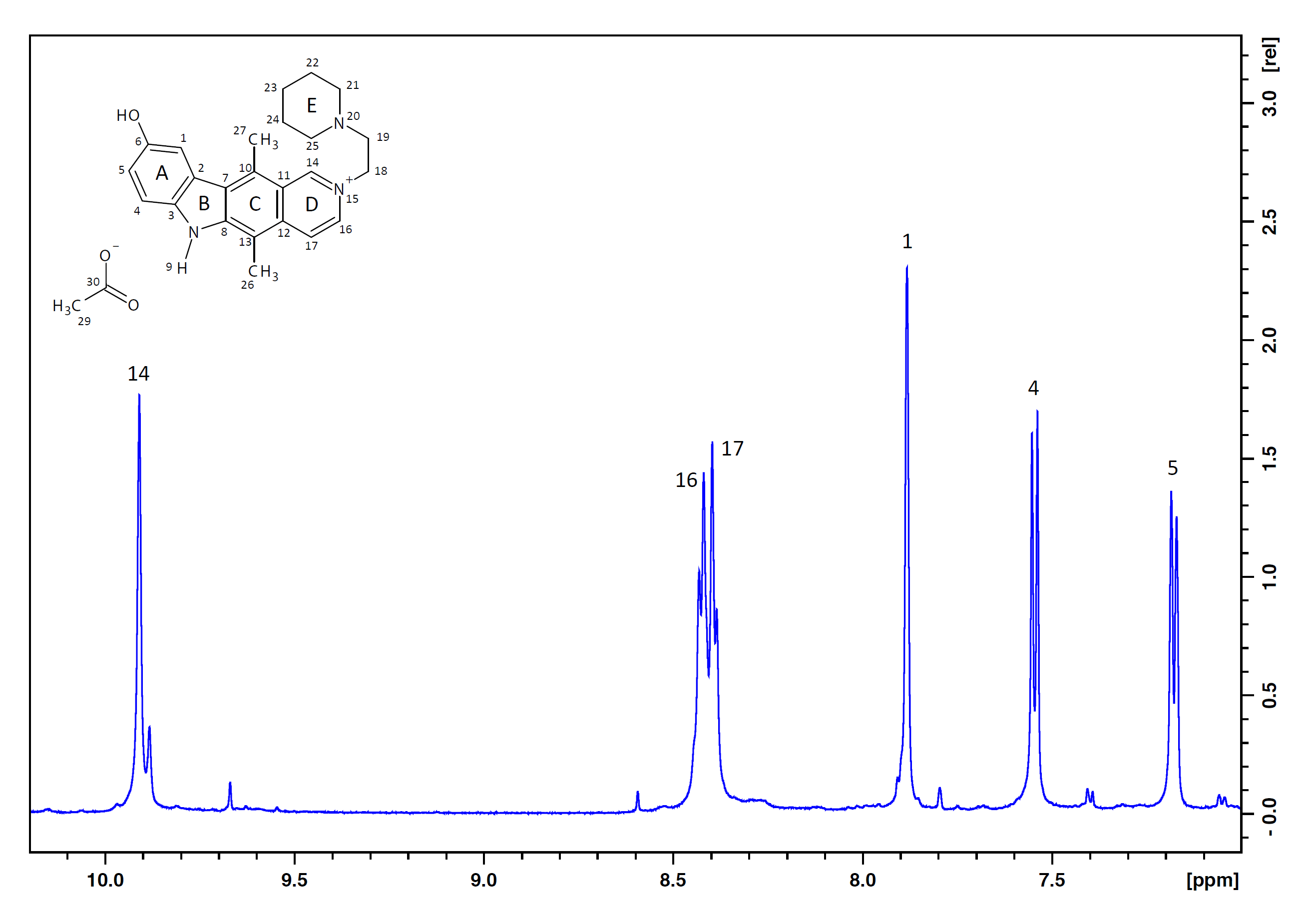
**Supplementary Figure 5.** ^1^H spectra of A9 in 100% DMSO with assignments, zoomed in from 6.9 to 10.3 ppm, known as the aromatic region.


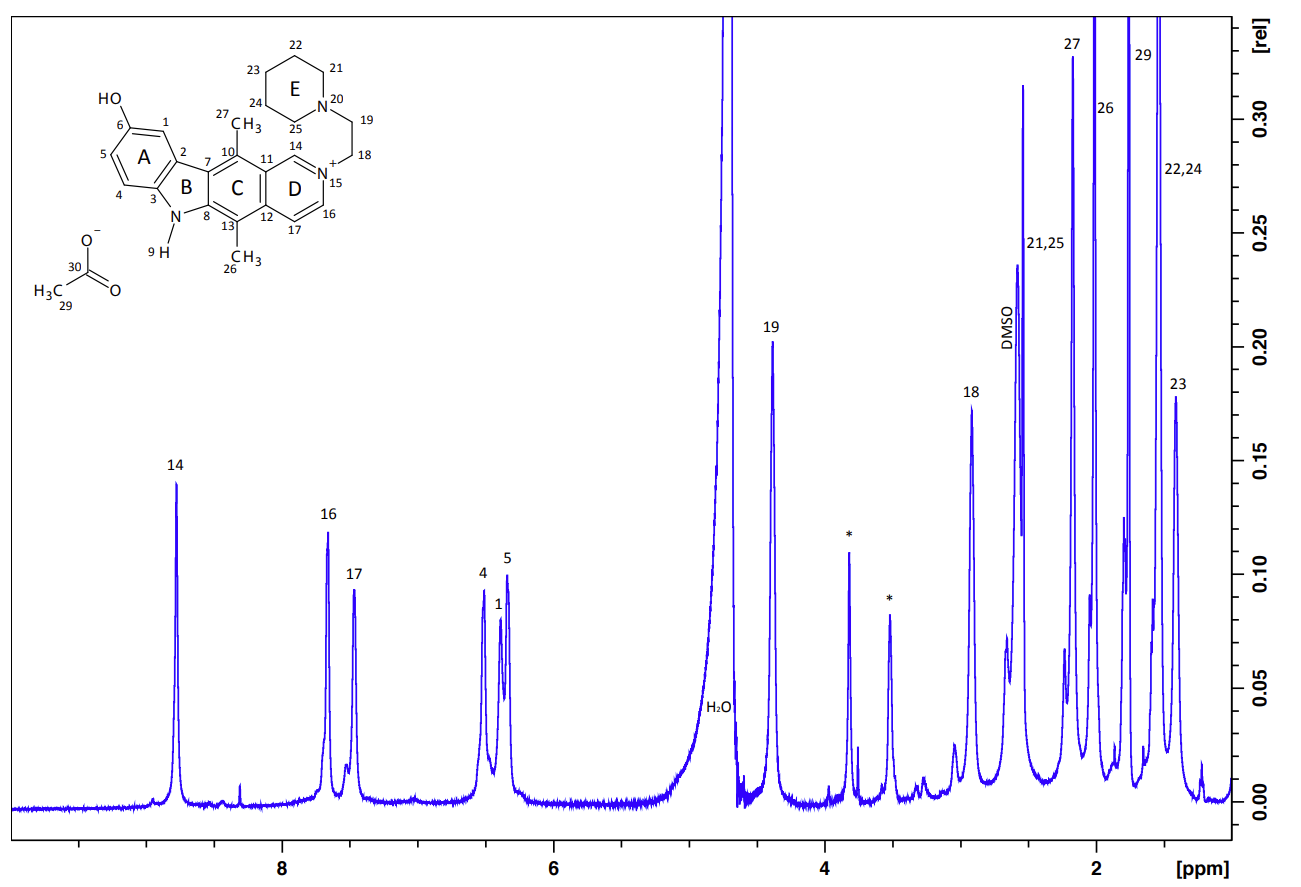
**Supplementary Figure 6.** ^1^H spectra of A9 in 1% DMSO with assignments. Asterisk (*) indicates impurity peak.


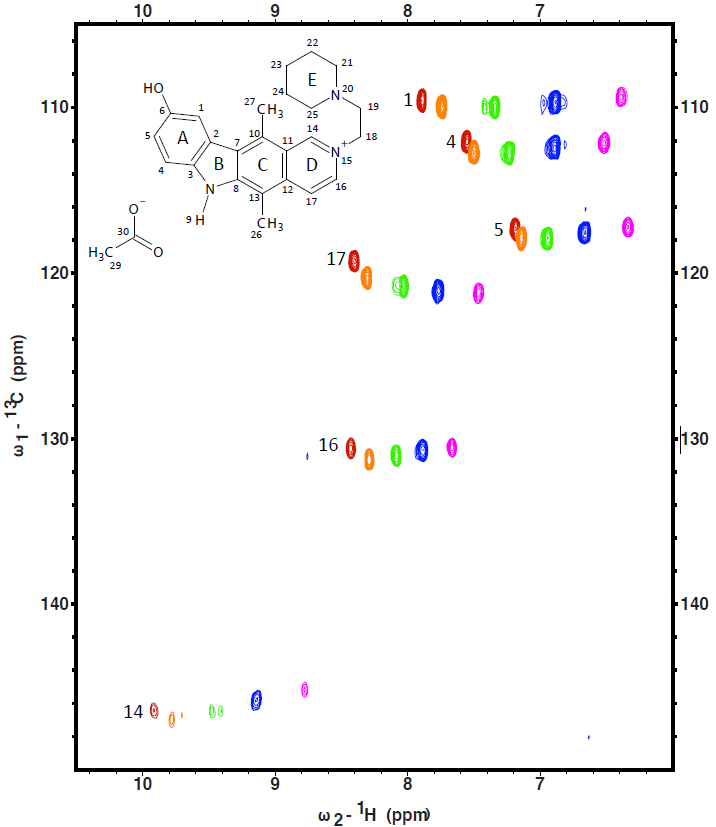


**Supplementary Figure 7.** ^13^C-^1^H HSQC spectra of A9 in decreasing percentage of deuterated DMSO. Red is 100%, orange is 75%, green is 50%, blue is 25%, and pink is 1% deuterated DMSO. Spectra processed in NMRFAM Sparky.


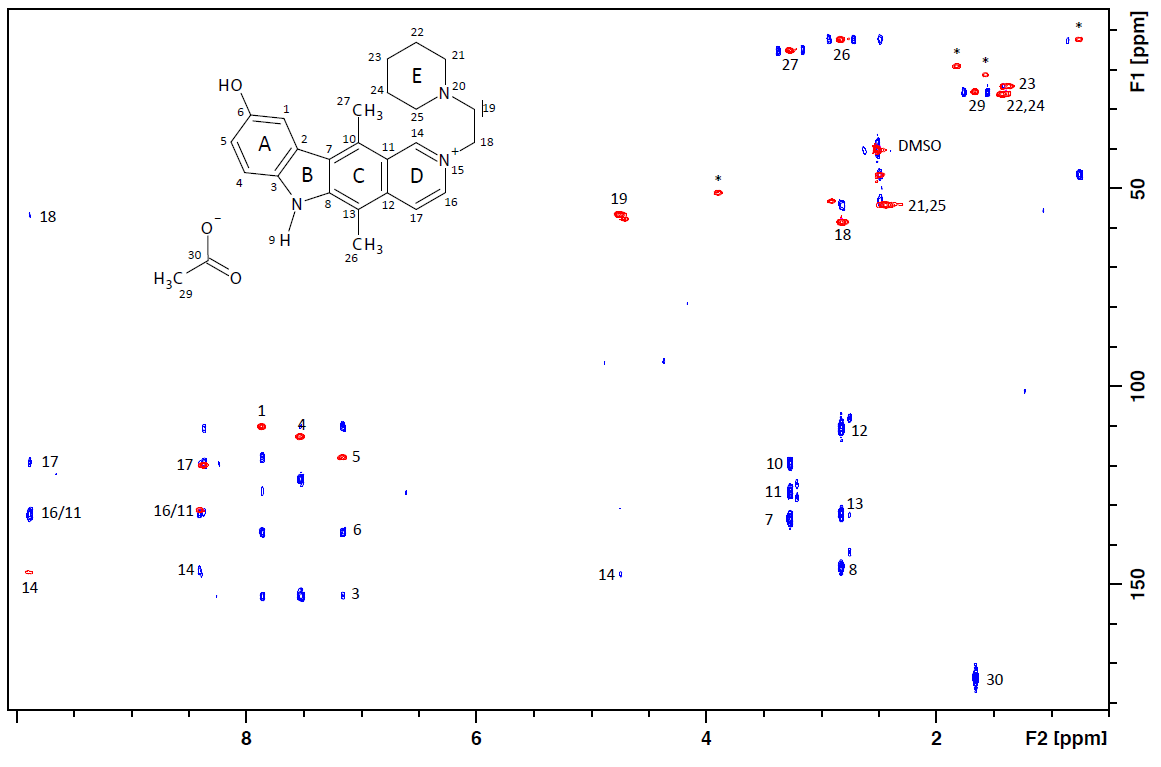
**Supplementary Figure 8.** Assigned ^1^H-^1^H HSQC (red peaks) and ^1^H-^1^H HMBC (blue peaks) overlay NMR of A9 in 100% DMSO. Asterisk (*) indicates impurity peak.


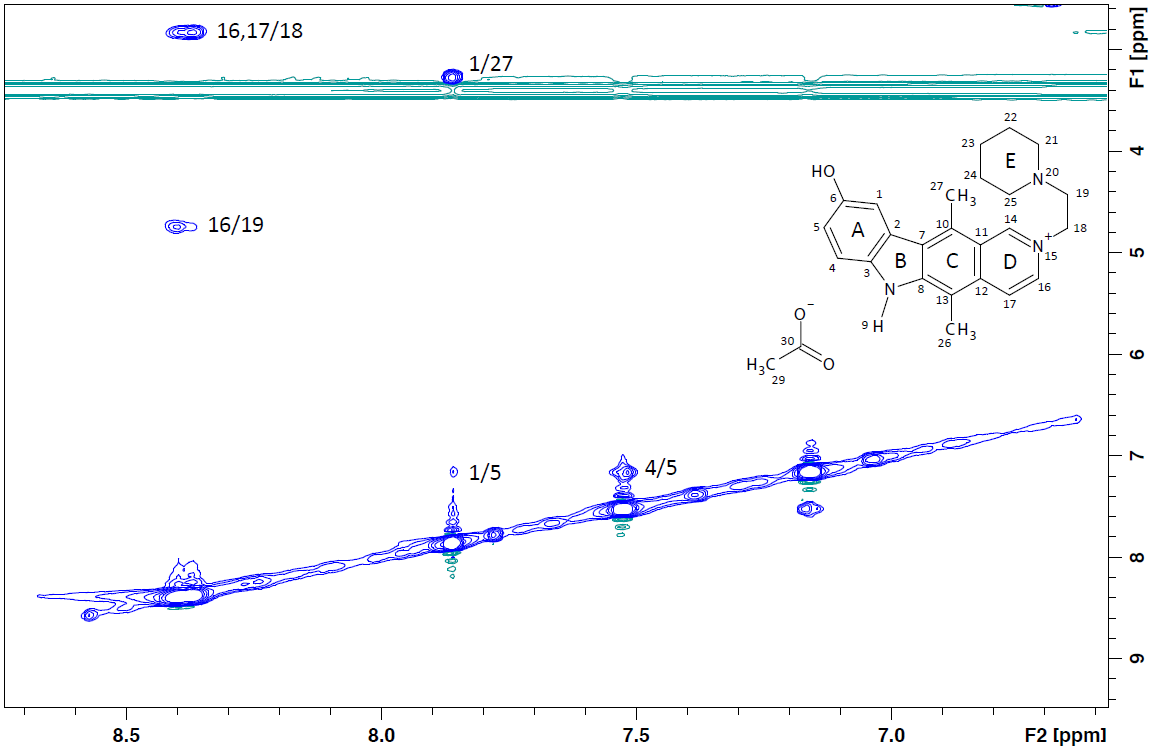
**Supplementary Figure 9.** Assigned ^1^H-^1^H NOESY NMR of A9 in 100% DMSO. Crosspeaks labeled as proton/carbon dimension assignment.


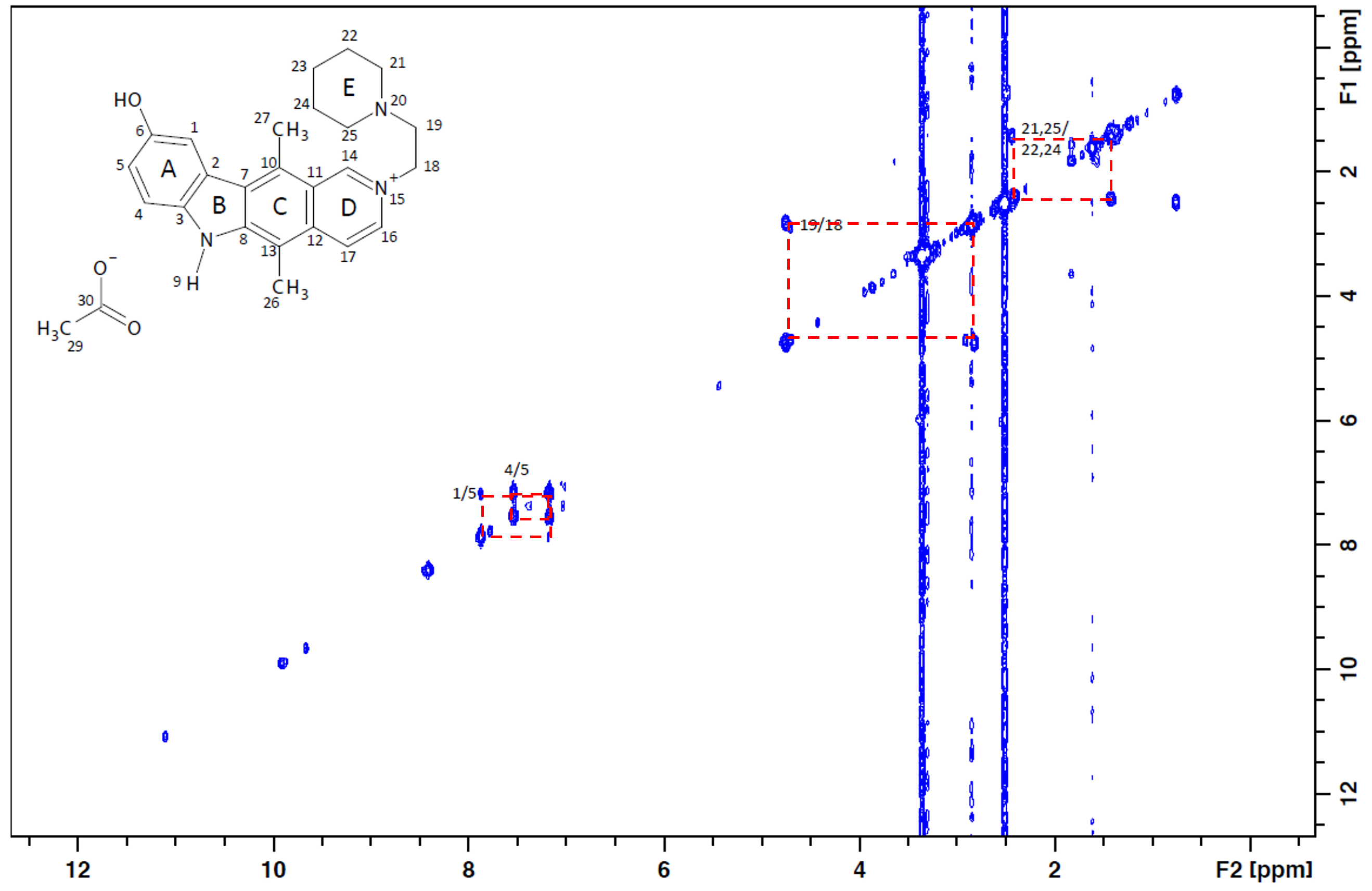
**Supplementary Figure 10.** Assigned ^1^H-^1^H COSY NMR of A9 in 100% DMSO. Crosspeaks labeled as proton/carbon dimension assignment.


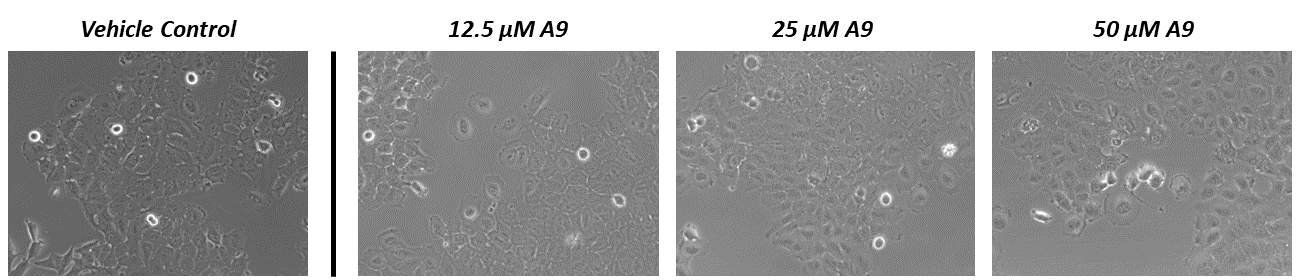
**Supplementary Figure 11.** Microscopy images of cytotoxicity assay with A9. H4 cells not treated with A9 in the leftmost panel (Vehicle Control). H4 cells treated with increasing concentrations of A9 (12.5, 25, and 50 μM, from left to right), show no significant toxicity.
